# Pure Air and Life

**Published:** 1892-07

**Authors:** 


### PURE AIR AND LIFE.

Exercise and pure air sustain us in our constant struggle against the poisons that we manufacture within ourselves, by driving the blood charged with oxygen more thoroughly through the tissue, thus quickening the breaking down of dead tissue into its safe and final waste products which make their exit through the natural channels. From this fact we may infer that the man of sedentary life requires of necessity pure air.

Pure air and exercise are equal forces acting in the same direction. They both get rid of waste, and with it the poisons in the system which are depressing various organs. We need not, therefore, be surprised when we are told by Sir D. Galton that after barracks were better ventilated the rations of the men had to be increased; or by "the pathetic story " of certain seamstresses whose work room was ventilated, and who then begged that the old state of things might be restored, as their appetites had increased beyond their earnings.

The same author gives another experience, illustrating the depressive effects of these poisons upon the functions of life. A medical man rather cruelly shut up some flies without food, some in foul air, others in pure air ; the pure air being constantly changed. To his surprise, the flies in the pure air died first, these dying from simple starvation; while the flies in the foul air died from poison, and with the tissues of their bodies inexhausted, indicating how the functions of life were carried on to the last where oxygen was available, but had been slowed and depressed by the presence of the poison, so that life was maintained longer in the foul than in the pure air.—*Popular Science Monthly.*

